# Autonomous STING signaling in Purkinje cells drives neurodegeneration independent of type I interferon

**DOI:** 10.1016/j.celrep.2025.116480

**Published:** 2025-10-29

**Authors:** Kun Yang, Miranda Dunn, Gustavo Torres-Ramirez, Nicole Dobbs, Vikram G. Shakkottai, Nan Yan

**Affiliations:** 1 Department of Immunology, UT Southwestern Medical Center, Dallas, TX, USA; 2 Department of Neurology and the O’Donnell Brain Institute, UT Southwestern Medical Center, Dallas, TX, USA; 3 Lead contact

## Abstract

STING signaling is emerging as a critical component of neurodegenerative diseases. While microglial STING-type I interferon (IFN-I) signaling is well-established, the role of STING signaling in neurons remains unclear. Here, we show that the STING protein is expressed in Purkinje cells of the cerebellum. Selective activation of STING signaling only in Purkinje cells using a conditional constitutively active N153S allele results in progressive neuronal loss and cerebellar atrophy, astrogliosis, but no microgliosis, leading to severe motor impairments in mice. Surprisingly, Purkinje cell STING activation does not induce IFN-I response. IFN-I receptor (*Ifnar1*) knockout also does not mitigate Purkinje cell STING-mediated neurodegeneration. Electrophysiological analyses reveal that Purkinje cell STING signaling reduces autonomous firing, which is essential for pace-making and neuronal function. Together, these findings demonstrate the physiological significance of IFN-independent STING function in Purkinje neurons and highlight its divergence from microglial STING signaling.

## In brief

Microglial STING-type I interferon signaling is known to play a role in neuroinflammation and neurodegeneration. Yang et al. find that STING is also expressed in Purkinje neurons. STING activation dysregulates the autonomous firing and pace-making function of Purkinje neurons independently of interferon signaling, leading to neurodegeneration and cerebellar ataxia.

## Graphical Abstract

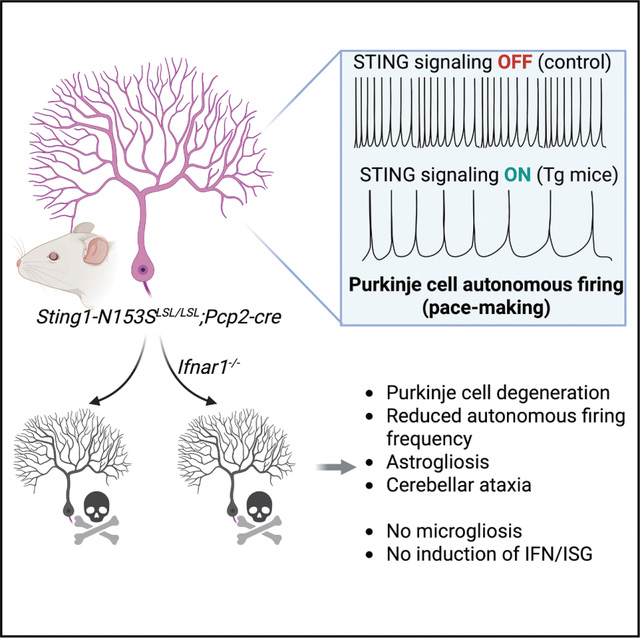

## INTRODUCTION

The cyclic guanosine monophosphate-adenosine monophosphate synthase (cGAS)-stimulator of interferon genes (STING) pathway is a crucial signaling pathway of the innate immune system, which mediates type I interferon (IFN-I) and inflammatory responses. This pathway has recently been implicated in various neurological diseases, and pharmacological inhibition of cGAS and STING is emerging as an attractive therapeutic strategy for treating neurodegeneration. In the brain, cGAS-STING activation mechanisms include aberrant exposure of mitochondrial or genomic DNA, which activates the cytosolic DNA sensor cGAS to produce cyclic guanosine monophosphate-adenosine monophosphate (cGAMP) that subsequently activates STING.^1–5^ Microglial cGAS-STING signaling plays an important role in neuroinflammation and neurodegeneration. However, how STING signaling causes neuronal loss and whether STING signaling in neuronal cells is also pathogenic remains largely unclear.

Studies of STING signaling in peripheral tissues have demonstrated that STING has both IFN-dependent and IFN-independent activities. In myeloid cells, including microglia, STING primarily drives IFN-I signaling, whereas in lymphoid cells and other cell types, STING signaling activities are largely IFN-independent and involve processes such as cell death, endoplasmic reticulum stress, autophagy, and proton channel function.^6–13^ The pattern of STING expression in different brain cell types, particularly non-glial cells, as well as STING signaling activities and physiological functions in neuronal cells, remain undefined. Here, we found endogenous STING expression in Purkinje cells, and STING activation selectively in Purkinje cells leads to drastic neuronal loss accompanied by movement disorder in mice. Genetic crossing reveals that neuronal STING signaling contributes to neurodegeneration independently of IFN-I. Electrophysiological studies reveal that IFN-independent STING activity disrupts Purkinje cell autonomous firing, which is essential for neuronal function and survival.

## RESULTS

### STING activation in Purkinje cells leads to severe neurological disease

**Figure 1.**
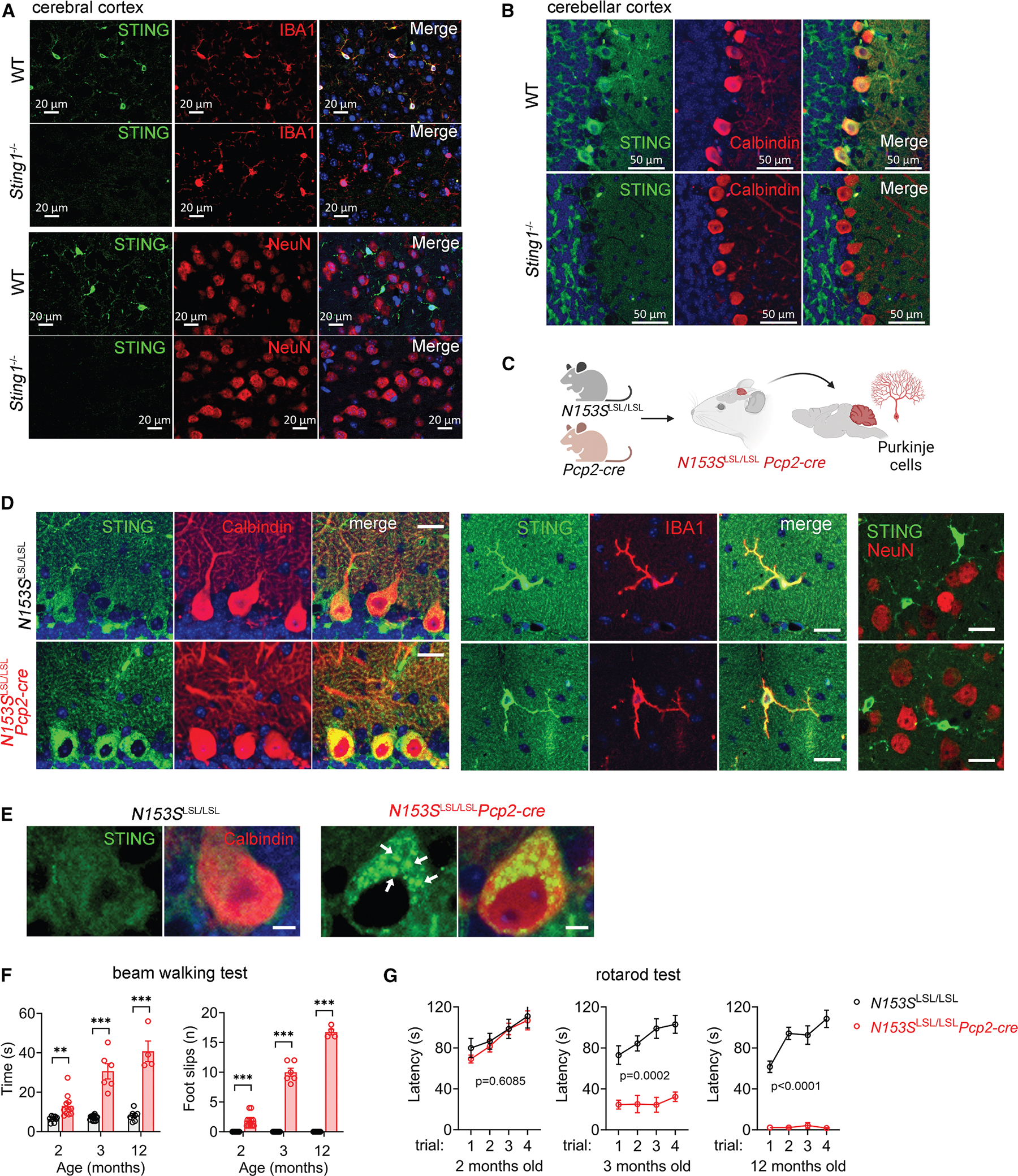
Purkinje cell-intrinsic STING signaling drives neurological disease (A and B) Immunofluorescence images of STING protein (green) in microglia (Iba1 in red), neurons (NeuN in red), and Purkinje cells (calbindin in red) in WT and *Sting1*^−/−^ mouse brains. Images are representative of at least *n* = 3 mice per genotype. Scale bars: 20 μm in (A) and 50 μm in (B). (C) A schematic graph showing the generation of N153SLSL/LSLPcp2-cre mice specifically expressing STING constitutively active N153S mutant in Purkinje cells. (D) Immunofluorescence images of STING expression (green) in Purkinje cells (calbindin in red), microglia (Iba1 in red), and neurons (NeuN in red) in 6-week-old *N153S*^LSL/LSL^*Pcp2-cre* and *N153S*^LSL/LSL^ mouse brains. Scale bars, 20 μm. Images are representative of at least *n* = 3 mice per genotype. (E) Immunofluorescence images of STING puncta (in green, denoted by white arrows) in Purkinje cells (calbindin in red) with low exposure time in 6-week-old *N153S*^LSL/LSL^*Pcp2-cre* and *N153S*^LSL/LSL^ mouse brains. Scale bar, 5 μm. Images are representative of at least 3 mice/genotype. (F and G) Beam walking and rotarod behavioral tests of *N153S*^LSL/LSL^ (*n =* 7–14) and *N153S*^LSL/LSL^*Pcp2-cre* (*n =* 4–11) mice at the indicated ages. Data are shown as mean ± SEM; **p < 0.01, ***p < 0.001, and ns, not significant; by two-tailed unpaired *t* test (E) or two-way ANOVA (F).

We first comprehensively analyzed STING protein expression in the mouse brain by co-staining STING and well-defined neural cell markers in wild-type mice (Figures 1A, 1B, and S1A). *Sting1*^−/−^ mice were used as negative controls to ensure the specificity of STING staining. In the cerebral cortex and hippocampus, STING was highly expressed in IBA1^+^ microglia and undetectable in NeuN^+^ neurons (Figures 1A, S1B, and S1B). In the cerebellum, STING protein was readily detected in calbindin^+^ Purkinje cells (Figure 1B). Very few microglia were present in the healthy cerebellum. Of note, public single-cell RNA sequencing (RNA-seq) analysis of mouse brains showed *Sting1* mRNA expression only in microglia, with low to undetectable expression in all neuronal cells, including Purkinje cells (Figures S1C and S1D). Homeostatic STING protein levels can be regulated at multiple post-translational levels, such as degradation by proteasomes and lysosomes.^2,14,15^ Therefore, our immunohistochemical (IHC) staining of STING protein provides more reliable information about STING expression pattern in the brain.

In various neurodegenerative conditions, the accumulation of endogenous STING proteins and the activation of STING signaling in neurons, including Purkinje cells, have been documented in both mouse models and human postmortem brain tissues.^16,17^ We next investigated whether autonomous STING signaling activation in Purkinje cells would interfere with their function and lead to neurological disorders. To precisely activate STING signaling only in Purkinje cells while avoiding introducing other cellular defects, we utilized a conditional allele of the STING constitutively active mutant N153S, which activates STING signaling independently of ligand binding.^18^ We crossed *Sting1-N153S* conditional knockin mice under the control of LoxP-Stop-LoxP (LSL) cassette (named *N153S*^LSL^ thereafter) to *Pcp2-cre* mice, thereby inducing STING signaling selectively in Purkinje cells (Figure 1C). Tissue-specific expression of STING-N153S was confirmed by an increase in STING protein level in Purkinje cells, but not in microglia or other neurons (Figure 1D). We also observed the formation of STING puncta and an increase in p-STING and p-TBK1 in *N153S*^LSL/LSL^*Pcp2-cre* Purkinje cells, indicating activation of STING signaling in these neurons (Figures 1E, S2A, and S2B).

*N153S*^LSL/LSL^*Pcp2-cre* mice were born at the expected Mendelian ratio and were indistinguishable from their *N153S*^LSL/LSL^ littermates before weaning. *N153S*^LSL/LSL^*Pcp2-cre* mice began to exhibit defects in coordination and motor function at 2 months of age, as assessed by the beam walking test (Figure 1F). The motor defects of *N153S*^LSL/LSL^*Pcp2-cre* mice progressively deteriorated with age, as demonstrated in both beam walking and rotarod tests (Figures 1F and 1G; Videos S1, S2, and S3). By 12 months of age, *N153S*^LSL/LSL^*Pcp2-cre* mice showed severely impaired balance and could barely stand on a stationary rotarod (Figure 1G; Video S4). Collectively, these results suggest that STING protein is naturally expressed in Purkinje cells and that autonomous STING activation in Purkinje cells is sufficient to drive neurological disease.

### STING signaling in Purkinje cells leads to neurodegeneration

**Figure 2.**
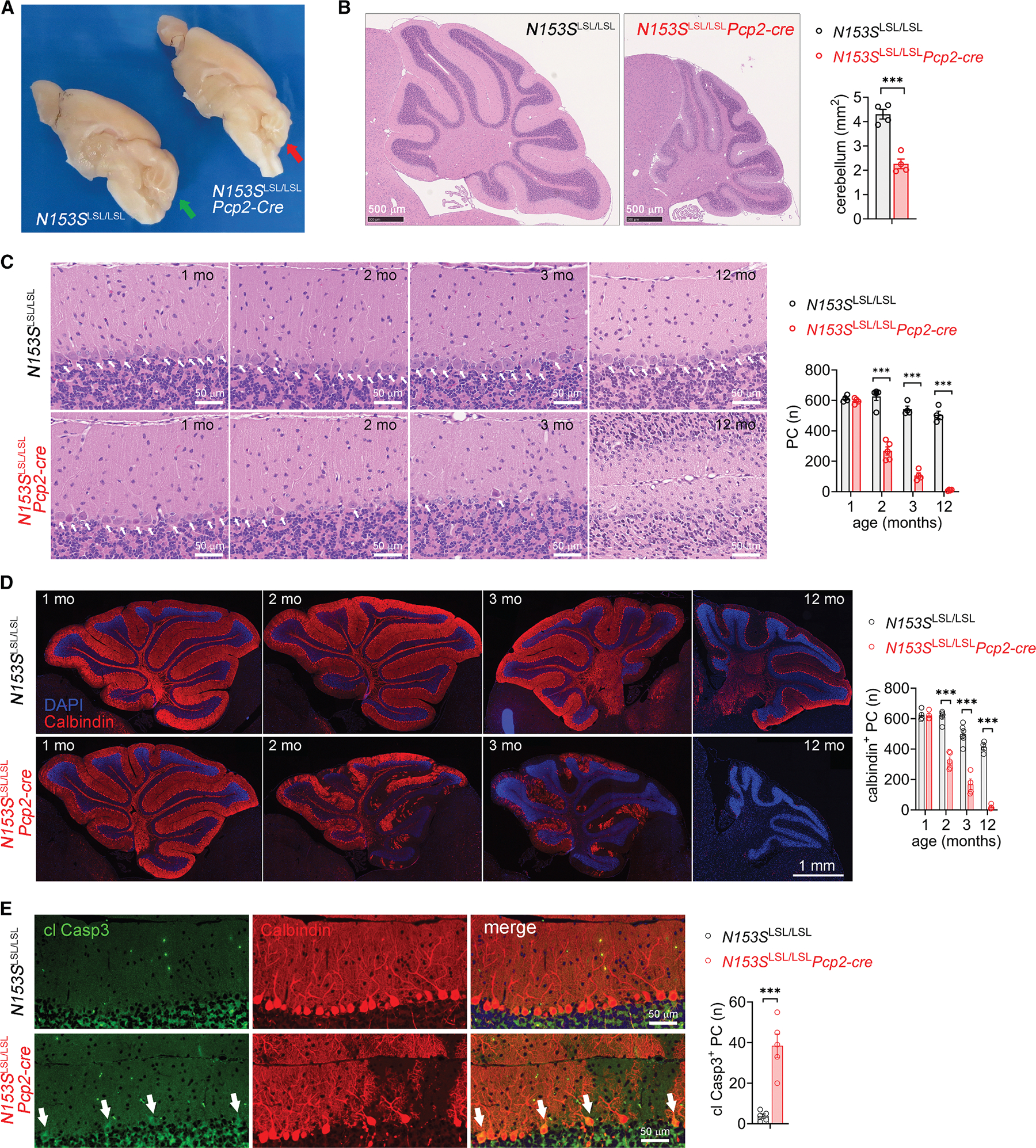
STING signaling in Purkinje cells leads to neurodegeneration (A) Images of mid-sagittally cut brains of 12-month-old *N153S*^LSL/LSL^*Pcp2-cre* and *N153S*^LSL/LSL^ littermate mice. Red arrow denotes cerebellar atrophy of *N153S*^LSL/LSL^*Pcp2-cre* mice. (B) Representative brain H&E staining of 12-month-old *N153S*^LSL/LSL^*Pcp2-cre* and *N153S*^LSL/LSL^ mice. Area of the cerebellar mid-sagittal section is quantified on the right. Data are shown as mean ± SEM; ***p < 0.001 by two-tailed unpaired *t* test. Scale bars, 500 μm. (C) Representative brain H&E staining of *N153S*^LSL/LSL^*Pcp2-cre* and *N153S*^LSL/LSL^ mice at indicated ages. Quantification of Purkinje cells is shown on the right. Data are shown as mean ± SEM; ***p < 0.001 by two-tailed unpaired *t* test. Scale bars, 50 μm. (D) Representative immunofluorescence images of Purkinje cells (calbindin in red) in *N153S*^LSL/LSL^*Pcp2-cre* and *N153S*^LSL/LSL^ mouse brains at the indicated ages. Nuclei were stained with DAPI in blue. Quantification of calbindin^+^ Purkinje cells per mid-sagittal section is shown on the right. Data are shown as mean ± SEM (*n* > 4 mice per genotype per time point). ***p < 0.001 by two-tailed unpaired *t* test. Scale bars, 1 mm. (E) Representative immunostaining of cleaved caspase 3 (in green) in 2-month-old *N153S*^LSL/LSL^*Pcp2-cre* and *N153S*^LSL/LSL^ mouse cerebella. Nuclei were stained with DAPI in blue. Quantification of cleaved Caspase 3 (cl Casp3)-positive Purkinje cells per mid-sagittal section is shown on the right. Data are shown as mean ± SEM (*n* = 5 mice per genotype). ***p < 0.001 by two-tailed unpaired *t* test. Scale bars, 50 μm.

We performed histopathological analysis of *N153S*^LSL/LSL^*Pcp2-cre* brain to investigate the pathogenesis of the neurological disease. At 12 months of age, *N153S*^LSL/LSL^*Pcp2-cre* mice exhibited notable cerebellar atrophy and significantly reduced cerebellar volume compared to *N153S*^LSL/LSL^ littermates (Figures 2A and 2B). H&E staining showed progressive loss of Purkinje cells in *N153S*^LSL/LSL^*Pcp2-cre* mice starting at 2 months of age (Figure 2C). Immunofluorescence staining of calbindin further showed almost complete loss of Purkinje cells and shrunken cerebellum in 12-month-old *N153S*^LSL/LSL^*Pcp2-cre* mice (Figure 2D). We also observed an anterior-to-posterior gradient in Purkinje cell degeneration of *N153S*^LSL/LSL^*Pcp2-cre* mice, with the majority of Purkinje cells lost in the anterior lobe by 3 months of age, whereas more than half remained in the posterior lobe (Figure S3A). Immunostaining of cleaved Caspase 3 showed that Purkinje cells underwent apoptosis in the *N153S*^LSL/LSL^*Pcp2-cre* cerebellum (Figure 2E). Additionally, we observed reduced density of granule cells in 12-month-old but not young (<3 months) *N153S*^LSL/LSL^*Pcp2-cre* mice (Figures S3B, S3C, and 2C), suggesting that granule cell loss is likely a secondary pathology. Both cleaved Caspase 3 and TUNEL staining showed cell death in both the granule cell layer and the molecular layer in 12-month-old *N153S*^LSL/LSL^*Pcp2-cre* mice (Figures S3D and S3E). These results suggest that cell-autonomous STING signaling in Purkinje cells leads to neuronal cell death and neurodegeneration.

### Ablation of IFN-I does not prevent STING-mediated neurodegeneration

**Figure 3.**
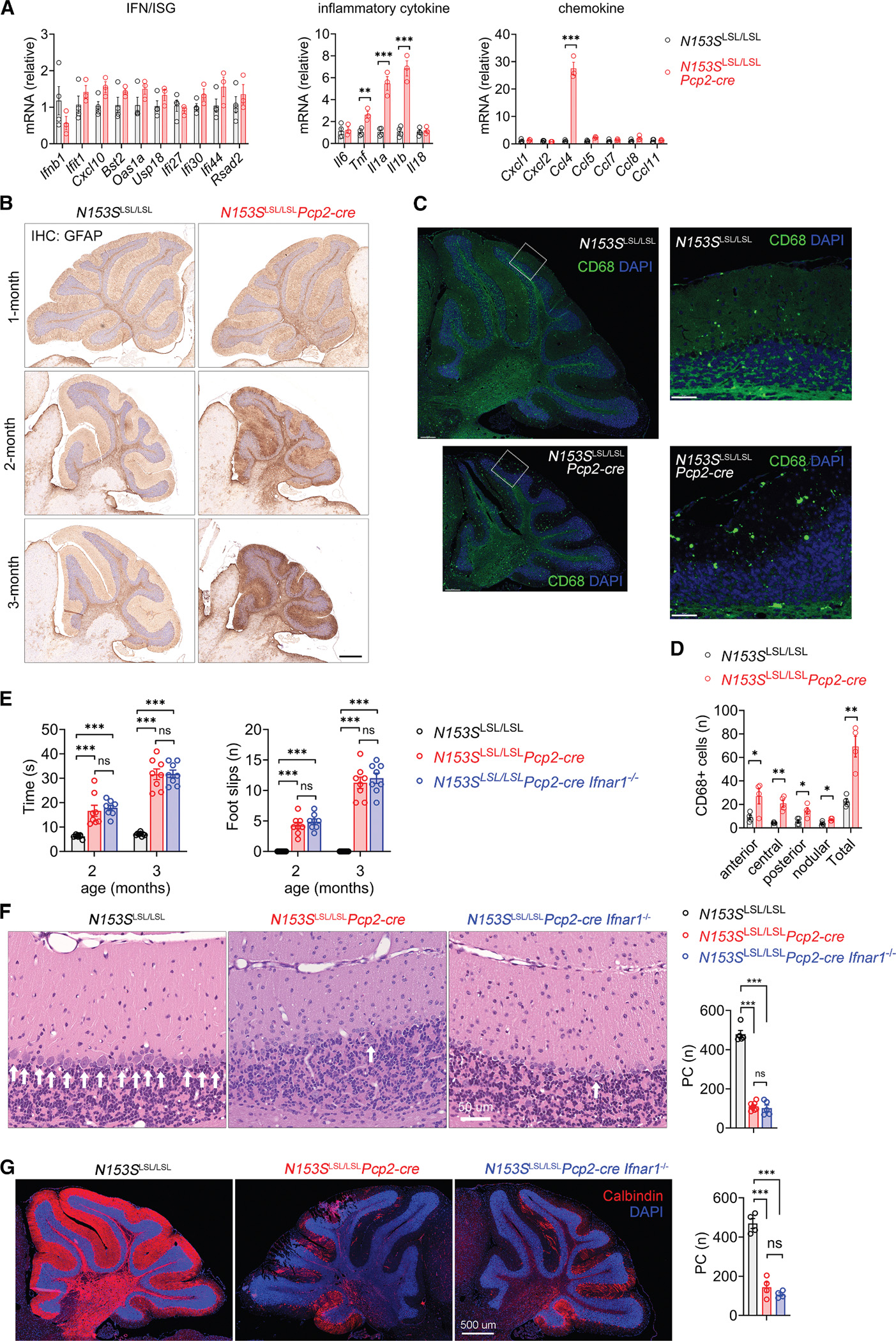
Type I IFN receptor ablation does not prevent Purkinje cell STING-mediated neurodegeneration (A) RT-qPCR analysis of 2-month-old *N153S*^LSL/LSL^ (*n* = 4) and *N153S*^LSL/LSL^*Pcp2-cre* (*n* = 3) mouse cerebella. Data are shown as mean ± SEM; two-tailed unpaired *t* test. **p < 0.01 and ***p < 0.001. (B) Representative IHC-3,3’-Diaminobenzidine (DAB) staining of astrocyte marker GFAP in *N153S*^LSL/LSL^ and *N153S*^LSL/LSL^*Pcp2-cre* mouse brains at the indicated ages. *n* = 4 per genotype per time point. Scale bars, 500 μm. (C) Representative immunofluorescence images of microglia activation marker CD68 (green) in 12-month-old *N153S*^LSL/LSL^*Pcp2-cre* and *N153S*^LSL/LSL^ mouse brains. Nuclei were stained with DAPI in blue. Note: cerebellar atrophy in *N153S*^LSL/LSL^*Pcp2-cre* brains. Scale bars: zoom-out (left panel), 200 μm; zoom-in (right panel), 50 μm. (D) Quantification of CD68^+^ microglia in anterior, central, posterior, and nodular lobes in (C). Data are shown as mean ± SEM (*n* = 4 mice per genotype). *p < 0.05 and **p < 0.01 by two-tailed unpaired *t* test. (E) Beam walking test of *N153S*^LSL/LSL^, *N153S*^LSL/LSL^*Pcp2-cre*, and *N153S*^LSL/LSL^*Pcp2-creIfnar1*^−/−^ mice at the indicated ages (*n* = 8/genotype). Data are shown as mean ± SEM; ***p < 0.001, ns, not significant, by two-tailed unpaired *t* test. (F) Representative cerebellar H&E staining of 3-month-old *N153S*^LSL/LSL^ (*n* = 5), *N153S*^LSL/LSL^*Pcp2-cre* (*n* = 7), and *N153S*^LSL/LSL^*Pcp2-creIfnar1*^−/−^ (*n* = 5) mice. Quantification of Purkinje cells is shown on the right. Data are shown as mean ± SEM; two-tailed unpaired *t* test; ***p < 0.001, ns, not significant. Scale bars, 50 μm. (G) Representative immunofluorescence images of Purkinje cells (calbindin in red) in 3-month-old *N153S*^LSL/LSL^, *N153S*^LSL/LSL^*Pcp2-cre*, and *N153S*^LSL/LSL^*Pcp2-creIfnar1*^−/−^ mice. Quantification of calbindin^+^ Purkinje cells is shown on the right (*n* = 4 mice per genotype). Data are shown as mean ± SEM; two-tailed unpaired *t* test; ***p < 0.001 and ns, not significant. Scale bars, 500 μm.

IFN-I and inflammatory responses are well-characterized downstream effector functions of STING signaling. To assess their activation, we first measured the mRNA expression levels of IFN-stimulated genes (ISGs), inflammatory cytokines, and chemokines in bulk cerebellar tissues from *N153S*^LSL/LSL^*Pcp2-cre* and *N153S*^LSL/LSL^ mice. Surprisingly, we did not observe any induction of IFN or ISGs in *N153S*^LSL/LSL^*Pcp2-cre* cerebella (Figure 3A). However, a number of inflammatory cytokines and chemokines were significantly upregulated in *N153S*^LSL/LSL^*Pcp2-cre* cerebella (Figure 3A). Damage or degeneration of Purkinje cells typically triggers reactive proliferation of Bergmann glia, which are regionally specialized radial astrocytes that support neuronal function in the cerebellum. ^19^ Immunostaining of the astrocyte marker GFAP showed increased astrogliosis in *N153S*^LSL/LSL^*Pcp2-cre* cerebellum beginning at 2 months of age (Figure 3B). Immunostaining of the microglia marker IBA1 did not reveal a significant difference in the number of microglia between the two genotypes (Figure S4A), although *N153S*^LSL/LSL^*Pcp2-cre* exhibited more CD68^+^ activated cells in the cerebellum than *N153S*^LSL/LSL^ (Figures 3C and 3D). We did not observe evidence of microglia activation in the cerebral cortex or hippocampus of *N153S*^LSL/LSL^*Pcp2-cre* brain (Figures S4B and 4C). To determine whether IFN-I signaling plays a role in cerebellar neurodegeneration in *N153S*^LSL/LSL^*Pcp2-cre* mice, we further crossed them to *Ifnar1*^−/−^ mice. *N153S*^LSL/LSL^*Pcp2-cre;Ifnar1*^−/−^ mice develop similar neurological disease as *N153S*^LSL/LSL^*Pcp2-cre* mice, as measured by the beam walking test (Figure 3E). H&E staining and immunofluorescence staining of calbindin also showed comparable Purkinje cell loss between *N153S*^LSL/LSL^*Pcp2-cre;Ifnar1*^−/−^ and *N153S*^LSL/LSL^*Pcp2-cre* mice (Figures 3F and 3G). These findings suggest an IFN-independent mechanism of STING-mediated neuronal cell death and neurodegeneration.

### STING activation disrupts Purkinje cell autonomous firing

**Figure 4.**
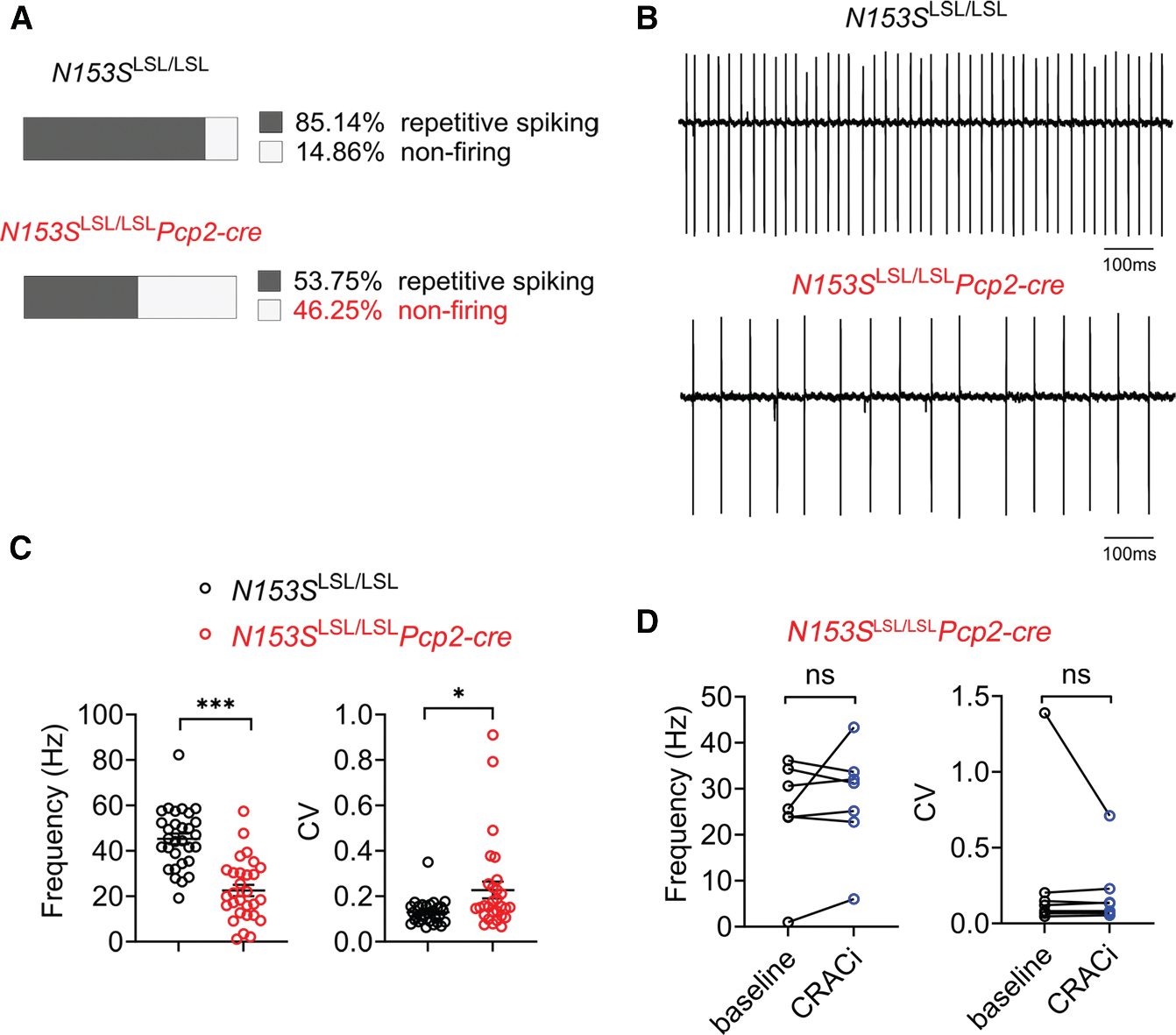
STING signaling disrupts Purkinje cells’ autonomous spiking (A) The percentage of Purkinje cells that lack spiking in 6-week-old *N153S*^LSL/LSL^ (*n* = 5) and *N153S*^LSL/LSL^*Pcp2-cre* (*n* = 3) mice brains. (B) Representative firing from *N153S*^LSL/LSL^ and *N153S*^LSL/LSL^*Pcp2-cre* Purkinje cells. (C) Firing frequency and coefficients of variation of *N153S*^LSL/LSL^ (29 cells from 5 mice) and *N153S*^LSL/LSL^*Pcp2-cre* (30 cells from 3 mice) Purkinje cells. Data were shown as mean ± SEM. *p < 0.05 and ***p < 0.001, unpaired Student’s *t* test. (D) Firing frequency and coefficients of variation of *N153S*^LSL/LSL^*Pcp2-cre* Purkinje cells (*n* = 7) treated with CRAC inhibitor GSK-7975A (CRACi, 20 μM) analyzed with a paired t test.

To further determine STING function in Purkinje cells, we performed electrophysiological studies in acute cerebellar slices of the mouse brain. Purkinje cells have intrinsic pace-making properties, allowing them to fire spontaneously at a baseline rate in the absence of synaptic input, which is normally extremely precise, with nearly uniform interspike interval duration.^20^ We chose 6-week-old *N153S*^LSL/LSL^*Pcp2-cre* mice that have not exhibited significant Purkinje cell loss and compared them to healthy Cre-negative littermate control mice. In noninvasive cell-attached recordings from cerebellar slices, wild-type Purkinje cells uniformly exhibited repetitive spiking (Figures 4A and 4B). In contrast, over 46% of Purkinje cells from *N153S*^LSL/LSL^*Pcp2-cre* mice showed no evidence of spiking (Figure 4A). The *N153S*^LSL/LSL^*Pcp2-cre* Purkinje cells that did exhibit repetitive spiking (~53%) displayed a significantly lower firing frequency that usually accompanies cell atrophy and ataxia^21,22^ (Figures 4B and 4C).

We further performed whole-cell patch-clamp recordings. The firing frequency in the *N153S*^LSL/LSL^*Pcp2-cre* mice remained significantly slower than in the wild-type mice (Figures S5A and S5B). In other models of ataxia, a reduction in firing frequency is often due to an increase in the amplitude and duration of the afterhyperpolarization potential (AHP) following the spike.^23,24^ Analysis of the spikes in *N153S*^LSL/LSL^*Pcp2-cre* revealed no alteration in the amplitude of the AHP, although the decay of the AHP was more rapid compared to wild-type mice (Figures S5C–S5E). Further, spikes from *N153S*^LSL/LSL^*Pcp2-cre* Purkinje cells had a significantly steeper upstroke and downstroke and correspondingly narrower half-width in the *N153S*^LSL/LSL^*Pcp2-cre* mice in spite of a reduction in firing frequency, suggesting that the more rapid depolarization in the interspike interval is mediated by a depolarizing conductance distinct from voltage-gated sodium channels (Figures S5F–S5H). The presence of a depolarizing current in the interspike interval is consistent with the observation that the spike threshold was significantly depolarized in *N153S*^LSL/LSL^*Pcp2-cre* compared to wild-type neurons. Since, normally, the depolarization in the interspike interval is driven entirely by voltage-gated sodium channels,^25^ an increase in available voltage-gated sodium current in the interspike interval would result in the spike threshold to be more hyperpolarized and an increase rather than the observed decrease in firing frequency. The analysis of spike characteristics suggests that a depolarizing conductance assumes a novel role in Purkinje neuron autonomous spiking by increasing the inactivation of the voltage-gated sodium current and resulting in less available sodium current in the interspike interval.

We next examined which STING activity is responsible for reduced Purkinje cell firing frequency in *N153S*^LSL/LSL^*Pcp2-cre* mice. STING-mediated IFN-I signaling acts through IFN-I receptor IFNAR-JAK-STAT pathway. STING activation can also release the ER calcium sensor stromal interaction molecule 1 (STIM1) and then activate the calcium-release-activated calcium (CRAC) channel.^26^ We first treated *N153S*^LSL/LSL^*Pcp2-cre* cerebellar slices with CRAC inhibitor GSK-7975A and observed no effect on Purkinje cell firing frequency (Figure 4D). Inhibiting IFN-I signaling with JAK inhibitor ruxolitinib also did not rescue the reduced firing frequency of *N153S*^LSL/LSL^*Pcp2-cre* Purkinje cells (Figure S5J). Together, these results suggest that STING signaling disrupts Purkinje cell autonomous firing and pace-making function that likely led to their degeneration.

### Dysregulated ion channels and synaptic receptors in *N153S*^LSL/LSL^*Pcp2-cre* cerebellum

**Figure 5.**
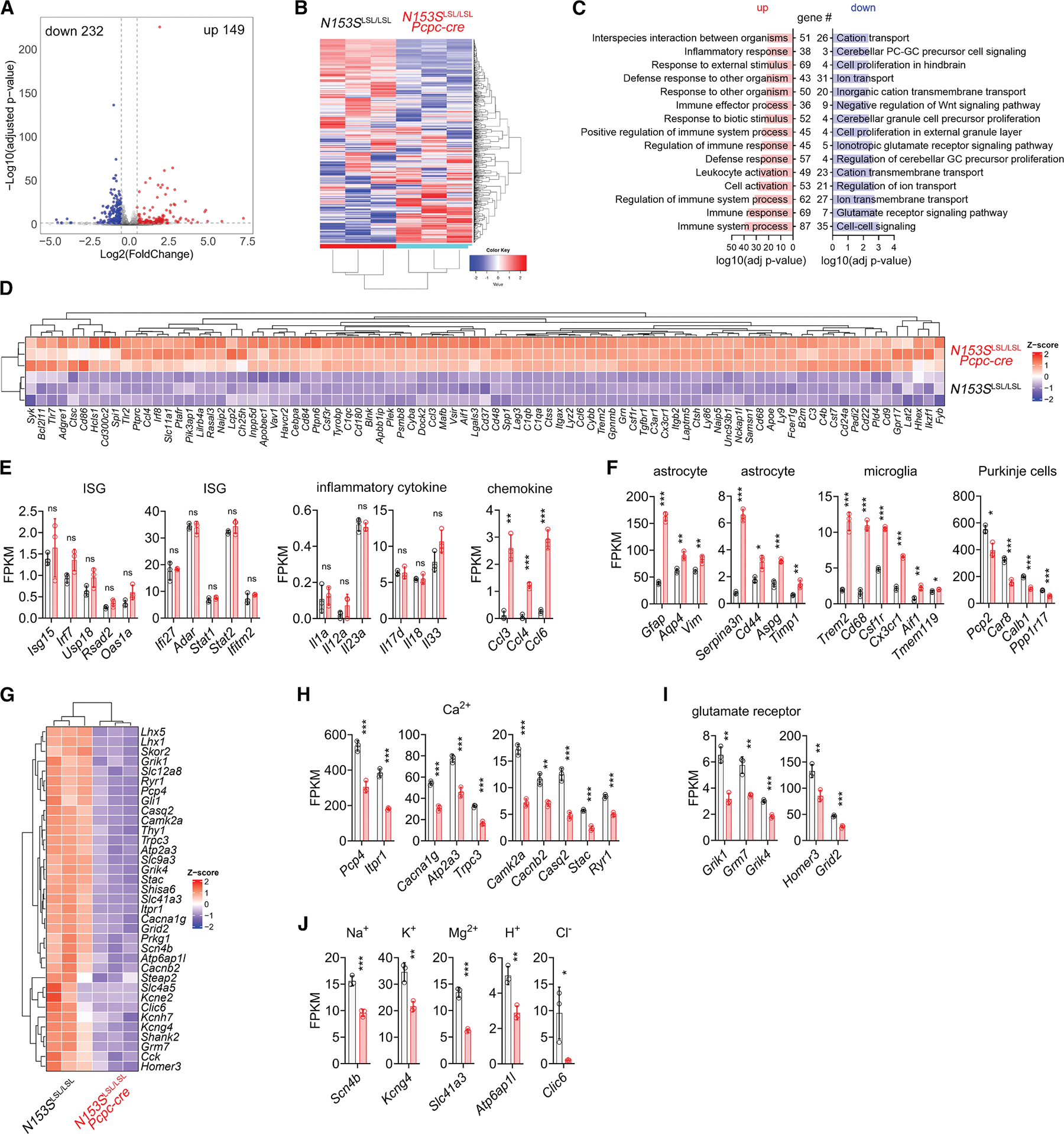
RNA-seq analysis of *N153S*^LSL/LSL^*Pcp2-cre* cerebellum (A) Volcano plot of DEGs between *N153S*^LSL/LSL^ (*n* = 3 mice) and *N153S*^LSL/LSL^*Pcp2-cre* (*n* = 3 mice) cerebella. (B) Heatmap of DEGs between *N153S*^LSL/LSL^ (*n* = 3 mice) and *N153S*^LSL/LSL^*Pcp2-cre* (*n* = 3 mice) cerebella. (C) GO analysis of DEGs between *N153S*^LSL/LSL^ (*n* = 3 mice) and *N153S*^LSL/LSL^*Pcp2-cre* (*n* = 3 mice) cerebella. (D) Heatmap of DEGs involved in immune response between *N153S*^LSL/LSL^ (*n* = 3 mice) and *N153S*^LSL/LSL^*Pcp2-cre* (*n* = 3 mice) cerebella. (E) Fragments per kilobase of exon per million mapped fragments (FPKM) of ISG, proinflammatory cytokines, and chemokines in *N153S*^LSL/LSL^ (*n* = 3 mice) and *N153S*^LSL/LSL^*Pcp2-cre* (*n* = 3 mice) cerebella. Data were shown as mean ± SEM. **p < 0.01, ***p < 0.001, and ns, not significant. Unpaired Student’s *t* test. (F) FPKM of cell-type-specific genes of astrocytes, microglia, and Purkinje cells in *N153S*^LSL/LSL^ (*n* = 3 mice) and *N153S*^LSL/LSL^*Pcp2-cre* (*n* = 3 mice) cerebella. (G) Heatmap of DEGs involved in ion transport between *N153S*^LSL/LSL^ (*n* = 3 mice) and *N153S*^LSL/LSL^*Pcp2-cre* (*n* = 3 mice) cerebella. (H–J) FPKM of genes involved in ion transport and glutamate receptor signaling in *N153S*^LSL/LSL^ (*n* = 3 mice) and *N153S*^LSL/LSL^*Pcp2-cre* (*n* = 3 mice) cerebella. Data were shown as mean ± SEM. *p < 0.05, **p < 0.01, ***p < 0.001, and ns, not significant. Unpaired Student’s *t* test.

To gain further insights into the pathogenic mechanism of Purkinje cell STING signaling, we performed bulk RNA-seq of *N153S*^LSL/LSL^*Pcp2-cre* and *N153S*^LSL/LSL^ cerebellar tissues at 2 months of age. We identified 149 upregulated and 232 downregulated genes in *N153S*^LSL/LSL^*Pcp2-cre* compared to *N153S*^LSL/LSL^ cerebellum (adjusted *p* ≤ 0.05, Log_2_ FoldChange≥0.5, Figure 5A). Gene ontology (GO) analysis of differentially expressed genes (DEGs) revealed that upregulated genes were predominantly enriched in immune response pathways, whereas downregulated genes were associated with ion transport and glutamate signaling (Figures 5B and 5C). Consistent with our quantitative reverse-transcription PCR (RT-qPCR) results (Figure 3A), RNA-seq data showed increased expression of a number of chemokines but not IFN or ISGs in *N153S*^LSL/LSL^*Pcp2-cre* cerebellum (Figures 5D and 5E). Additionally, we observed upregulation of astrocytic markers and microglial activation markers, including CD68, in *N153S*^LSL/LSL^*Pcp2-cre* cerebellum, while Purkinje cell-specific genes were downregulated (Figure 5F), which was in line with astrogliosis and Purkinje cell loss observed in histopathological analysis (Figures 2C, 2D, 3B, and 3C). Notably, we observed significant downregulation of numerous genes involved in calcium-related channels and transporters, other ion channels, and glutamate receptors (Figures 5G–5J). Dysregulated ion channels and synaptic receptors are consistent with the electrophysiological defects in Purkinje cells, further supporting an important role of STING in Purkinje cell function.

## DISCUSSION

Here, we demonstrate STING expression in Purkinje cells and STING activity in disrupting Purkinje cell autonomous firing, leading to neurodegeneration and movement disorder in mice. Our findings reveal a striking dichotomy of STING expression between the cerebrum and the cerebellum: STING protein level is high in microglia, not neurons, in the cerebrum, whereas in the cerebellum, STING protein level is high in Purkinje neurons, not glial cells. Interestingly, in human postmortem tissues from individuals with amyotrophic lateral sclerosis, increased STING expression has been observed in vulnerable cortical and spinal motor neurons.^17^ Similarly, neuronal STING expression is induced under inflammatory conditions in both human multiple sclerosis and in the mouse model of experimental autoimmune encephalomyelitis (EAE).^27^ Neuron-intrinsic STING signaling was also reported in mouse models of several lysosomal storage disorders.^16^ These observations suggest that, in addition to glial STING driving neuroinflammation, neuronal STING may also respond to neuroinflammatory stress and then more directly contribute to neurodegeneration in disease contexts. In our model, STING signaling triggered by a constitutively active STING allele causes Purkinje cell death by apoptosis, progressive neurodegeneration, and severe cerebellar neuropathology. Notably, genetic and electrophysiological studies collectively demonstrate an IFN-independent function of STING in membrane depolarization that disrupts autonomous firing and survival of Purkinje cells.

Purkinje cells are large neurons in the cerebellum, central to motor function and coordination.^28^ They are highly vulnerable to cell death in response to diverse genetic defects and environmental stresses.^29^ Purkinje cell pathology has been observed in many movement disorders, such as cerebellar ataxia, the causes of which are diverse.^22^ However, patients often present similar clinical and neuropathological features, including abnormal gait, poor coordination, speech impairment, Purkinje cell loss, thinning of molecular layers, and cerebellar atrophy. Our *N153S*^LSL/LSL^*Pcp2-cre* mice present many of these behavioral features, suggesting that Purkinje cell STING signaling is a previously unappreciated modulator of cerebellar neuropathology. An anterior-to-posterior pattern of Purkinje cell loss has been observed in various forms of cerebellar degeneration.^30–34^ This pattern may be influenced by factors such as metabolic demand, vascular supply, and susceptibility to specific toxins, depending on the underlying defects or insults. The gradient of degeneration from anterior to posterior in our mouse model could be related to spatial differences in STING signaling strength or intrinsic protective mechanisms of Purkinje cells, which need further investigation.

The dichotomy between microglial and neuronal STING functions resembles those in macrophages and T lymphocytes.^35^ Both microglia and macrophages are myeloid cells, and STING signaling is predominantly IFN-dependent. In contrast, STING signaling in both T lymphocytes and Purkinje neurons is IFN-independent, and STING activation leads to both T cell death^35^ and Purkinje cell loss (this study). Interestingly, T lymphocytes and neurons are developmentally divergent, but both rely on ion channels to function. The precise mechanisms of STING-mediated cell death in T lymphocytes and Purkinje cells remain to be elucidated.

One possibility is STING-mediated autophagy promoting neuronal cell death. We observed an overall increase in total LC3 levels in Purkinje cells from mutant mice. However, we were unable to detect distinct LC3 puncta, likely due to the limitations of immunostaining for endogenous LC3, as opposed to using transgenic reporter lines such as GFP-LC3 mice. In the EAE mouse model, neuronal STING signaling promotes autophagy-dependent neuronal cell death.^27^ Whether STING-mediated autophagy contributes to Purkinje cell loss requires further investigation. The increased expression of certain chemokines in cerebellar tissues may reflect astrogliosis secondary to Purkinje cell death.

Another possibility is STING proton channel function dysregulating ion channels in Purkinje neurons. Purkinje cells exhibit spontaneous firing in the absence of synaptic input, a property driven by their intrinsic membrane properties. This spontaneous activity is crucial for maintaining cerebellar function and is primarily regulated by various ion channels (Na^+^, K^+^, and Ca^2+^).^28,36^ We showed that STING activation in Purkinje cells reduces their firing frequency, which cannot be restored by CRAC channel inhibitor or JAK1/2 inhibitor treatment. A recently discovered function of STING is its role as a proton channel, which causes proton leakage from acidified intracellular organelles, such as the Golgi apparatus and endolysosomes.^12,13^ The STING proton channel function is also required for STING-mediated non-canonical autophagy and cell death.^13^ Therefore, another possibility is that STING-mediated proton leakage may directly or indirectly (e.g., via proton-gated ion channels) affect plasma membrane potential. Consistent with this possibility, our RNA-seq analysis of cerebellar tissue revealed broad downregulation of genes involved in ion transport. These alterations are likely secondary to dysregulation of ion homeostasis, as STING is unlikely to directly regulate ion transport-related genes. Furthermore, we cannot rule out the possibility that these transcriptional changes are driven by drastic shifts in cell type composition in cerebellar tissue, such as Purkinje cell loss and astrogliosis.

In summary, this study demonstrates that Purkinje cell STING signaling activation is sufficient to cause neurodegeneration and movement disorder independent of type I IFN signaling. We provided direct genetic and pathophysiological evidence for STING expression and activity in Purkinje cells; whether this mechanism is also applicable to other neurons remains to be defined. Although there is ample evidence for microglial STING-mediated neuroinflammation, the dichotomy of STING expression in the cerebrum and cerebellum as well as STING activities in myeloid and non-myeloid cells should be considered to fully understand the pathogenesis of neurodegenerative disease.

### Limitations of the study

This study suggests an IFN-independent activity of STING in Purkinje cells that is neuropathogenic. However, due to the lack of molecular tools to perturb each IFN-independent activity of STING as well as the lack of a transducible Purkinje cell culture system, the precise STING activity and molecular mechanism require further study. We performed bulk RNA-seq analysis of cerebellar tissue. Single nuclear RNA-seq of enriched Purkinje cells from cerebellar tissue should provide more insights into STING-mediated gene signature and subpopulation changes in Purkinje cells. An anterior-to-posterior gradient in Purkinje cell degeneration was observed in transgenic mice. Co-staining of Aldolase C and calbindin on coronal sections would provide additional information regarding the differential vulnerability of Zebrin-positive versus Zebrin-negative STING-expressing Purkinje cells.

### RESOURCE AVAILABILITY

#### Lead contact

Further information and requests for resources and reagents should be directed to and will be fulfilled by the lead contact, Dr. Nan Yan (nan.yan@utsouthwestern.edu).

#### Materials availability

All materials generated in this study are available from the lead contact.

#### Data and code availability

All data generated or analyzed during this study are included in the article or its supplemental information files. The accession number for RNA-seq datasets is GEO: GSE300185.

## STAR★METHODS

### METHOD DETAILS

#### Animals

*Sting1 N153S*^LSL^ mice were kind gifts from Dr. Jonathan J. Miner (University of Pennsylvania, Philadelphia, PA).^18^ B6.129-Tg(Pcp2-cre)2Mpin/J (*Pcp2-cre*, JAX Strain #: 004146), B6(Cg)-*Ifnar1*^*tm1.2Ees*^/J (*Ifnar1*−*/*−, JAX Strain #:028288), C57BL/6J (JAX stock #000664) mice were purchased from Jackson Laboratories. *Sting1*^−/−^ were obtained from Glen N. Barber (University of Miami).^37^ Both male and female animals were used in the study, and sex variable did not affect experimental outcomes. All mice were housed in specific pathogen-free barrier facilities at UT Southwestern Medical Center. The animal protocol was approved by the Institutional Animal Care and Use Committee at UT Southwestern Medical Center (APN 2017–101968).

#### Mouse motor function tests

Beam walking test was adopted from a previous study.^38^ Briefly, mice were trained to walk along a wood beam (3.2 cm wide, 60 cm long) elevated 30 cm above the bench to reach a goal box at the other end of the beam one day before test. Mice were tested the next day on a wood beam (1.6 cm wide, 60 cm long) elevated 30 cm above the bench. Mice were allowed to walk from one end of the beam to a goal box at the other end. The time on beam and the number of foot slips were measured and counted.

Rotarod test was performed per the Standard Operating Procedure of The Jackson Laboratory Mouse Neurobehavioral Phenotyping Facility (SOP-JAX-MNBF_ROT Version 1.00). In brief, mice were left undisturbed to acclimate for 60 min prior to testing. The mice were placed on the rotarod (Panlab, Harvard Apparatus), starting at a speed of 4 RPM, gradually accelerating over 300 s to reach 40 RPM. The latency time mice maintained their balance on the rotarod prior to falling off of the rod was recorded. Mice were tested in 4 trials with intertrial interval of about 20 min.

#### Histopathology, immunohistochemistry

H&E staining and TUNEL staining of mouse tissues were performed in UT Southwestern Medical Center Histo Pathology Core. Fluorescent immunohistochemistry and immunohistochemistry staining using 3,3′-Diaminobenzidine (IHC-DAB) were performed in UT Southwestern Medical Center Tissue Management Shared Resource core. Primary antibodies used for immunohistochemistry are as follows: TMEM173/STING Polyclonal antibody (Proteintech, catalog # 19851–1-AP, 1:500 dilution); Anti-NeuN Antibody (Millipore Sigma, catalog # MAB377, 1:400 dilution); Anti-Iba1/AIF1 Antibody (Millipore Sigma, catalog # MABN92, 1:400 dilution); Monoclonal Anti-Calbindin-D-28K antibody (Sigma-Aldrich, catalog #C9848, clone CB-955,1:1000 dilution); Anti-CD68 antibody (Abcam, catalog # ab283654, 1:100 dilution); Cleaved Caspase-3 (Asp175) Antibody (Cell Signaling, catalog # 9661, 1:200 dilution); Phospho-STING (Ser365) (D1C4T) Rabbit mAb (Cell Signaling, catalog # 62912, 1:50 dilution); Phospho-TBK1/NAK (Ser172) (D52C2) XP Rabbit mAb (Cell Signaling, catalog # 5483, 1:50 dilution); Anti-GFAP Antibody (SigmaMillipore, catalog # MAB360, 1:400 dilution); Anti-LC3B antibody (Abcam, catalog # ab192890, 1:500 dilution). Fluorophore-conjugated secondary antibodies were used as follows: Goat anti-Rabbit IgG (H + L) Highly Cross-Adsorbed Secondary Antibody, Alexa Fluor Plus 488 (Thermo Fisher, Catalog # A32731, 1:200 dilution); Donkey anti-Mouse IgG (H + L) Highly Cross-Adsorbed Secondary Antibody, Alexa Fluor 546 (ThermoFisher, Catalog # A10036, 1:200 dilution); Donkey anti-Mouse IgG (H + L) Highly Cross-Adsorbed Secondary Antibody, Alexa Fluor 647 (Thermo Fisher, Catalog # A-31571, 1:500 dilution). Nuclei were counterstained with DAPI. Immunofluorescence images were acquired using a Zeiss LSM 780 or 880 confocal microscope (Zeiss) in UT Southwestern Medical Center Live Cell Imaging Core or using Zeiss Axioscan.Z1 in UTSW Whole Brain Microscopy Facility. Calbindin immunoreactive Purkinje cells were quantified using QuPath-0.3.2 software.

#### RNA isolation and RT-qPCR

Total RNA was isolated from homogenized cerebellar tissues using TRI reagent (MilliporeSigma) per manufacturer’s instruction, and cDNA were synthesized with iScript cDNA Synthesis Kit (Bio-Rad). iTaq Universal SYBR Green Supermix (Bio-Rad) was used to quantify mRNA expression with CFX96 Real-Time PCR Detection System.

#### Patch-clamp electrophysiology

##### Solutions and reagents

Artificial CSF (aCSF) contained the following (in mmol/L): 125 NaCl, 3.8 KCl, 26 NaHCO3, 1.25 NaH2PO4,2 CaCl2, 1 MgCl2, 10 dextrose. Pipettes were filled with an internal recording solution containing the following (in mmol/L): 119 K Gluconate,2 Na gluconate, 6 NaCl, 2 MgCl2, 0.9 EGTA, 10 HEPES,14 Tris-phosphocreatine, 4 MgATP, 0.3 tris-GTP, pH 7.3, osmolarity 290 mOsm. JAK inhibitor Ruxolitinib (MedChemExpress, Cat. No.: HY-50856) was used at 10 μM for assessing somatic spiking in Purkinje Cells. CRAC inhibitor GSK-7975A (MilliporeSigma) was used at 20 μM for pharmacological experiments as well.

##### Acute slice preparation

Mice were anesthetized via isoflurane, decapitated, and brains were removed for slice preparation. Slices were prepared at physiological temperature (as described previously^32^) in prewarmed aCSF (33°C–36°C) and cut with a vibratome (Leica Vt1200s) and a ceramic blade. Sections were cut at a thickness of 300 μm and incubated in carbogen-bubbled (5% CO_2_, 95% O_2_) aCSF at 33°C–36°C for 45 min after sectioning.

##### Patch-clamp recordings

Patch-clamp recordings were performed as described previously.^32^ Cell-attached and whole-cell recordings were performed at 33°C in carbogen-bubbled aCSF at a flow rate of 2–3 mL/min less than 5 h after slice preparation. Recordings were performed using an Axopatch 200B amplifier, Digidata 1440A interface, and pClamp-10 software (MDS analytical technologies, Sunnyvale, CA). Data were acquired at 100 kHz in the fast current-clamp mode of the amplifier. Cells were rejected for whole-cell analysis if the series resistance changed by more than 20% or if it exceeded 15 MΩ. For recordings involving pharmacologic agents, baseline data were acquired for 4–5 min before perfusing with the agents and recording for another 4–5 min.

#### RNA sequencing

Two-month-old *N153S*^LSL/LSL^*Pcp2-cre* and *N153S*^LSL/LSL^ mice (3 mice per genotype) were perfused with ice-cold PBS and cerebella were collected and snap-frozen in liquid nitrogen. Total RNA was extracted from frozen cerebellar tissue using TRI Reagent (MilliporeSigma) per manufacturer’s instructions. The quality of RNA samples was examined using Agilent 2100 Bioanalyzer (Agilent). Total RNA was provided to Novogene (CA, USA) for poly A enrichment and mRNA library preparations. Library sequencing was performed on NovaSeq X Plus Series (PE150) platform. Analysis of raw data was done using Astrocyte RNASeq Analysis Workflow at BioHPC facility of UT Southwestern Medical Center. Briefly, trimmed Fastq files are aligned to the reference genome (mouse GRCm39) using STAR. Features (genes, transcripts and exons) are counted using featureCounts and StringTie using the Gencode feature table. Basic pairwise differential expression analysis is performed using EdgeR and DESeq.

### QUANTIFICATION AND STATISTICAL ANALYSIS

Graphpad Prism was used for statistical analysis. Statistical tests performed were indicated in figure legends. *p* values of less than 0.05 were considered statistically significant. Significant differences are indicated by *p < 0.05, **p < 0.01, ***p < 0.001.

## Supplementary Material

1

2

3

4

5

SUPPLEMENTAL INFORMATION

Supplemental information can be found online at https://doi.org/10.1016/j.celrep.2025.116480.

## Figures and Tables

**KEY RESOURCES TABLE:** 

REAGENT or RESOURCE	SOURCE	IDENTIFIER

Antibodies		

TMEM173/STING Polyclonal antibody	Proteintech	catalog # 19851-1-AP; RRID:AB_10665370
Anti-NeuN Antibody	MilliporeSigma	catalog # MAB377; RRID:AB_2298772
Anti-Iba1/AIF1 Antibody	MilliporeSigma	catalog # MABN92; RRID:AB_10917271
Monoclonal Anti-Calbindin-D-28K antibody	MilliporeSigma	catalog #C9848; RRID:AB_476894
Anti-CD68 antibody	Abcam	catalog # ab283654; RRID:AB_2922954
Cleaved Caspase-3 (Asp175) Antibody	Cell Signaling	catalog # 9661; RRID:AB_2341188
Anti-GFAP Antibody	MilliporeSigma	catalog # MAB360; RRID:AB_11212597
Phospho-TBK1/NAK (Ser172) (D52C2) XP^®^ Rabbit mAb	Cell Signaling	catalog # 5483; RRID:AB_10693472
Phospho-STING (Ser365) (D1C4T) Rabbit mAb	Cell Signaling	catalog # 62912; RRID:AB_2799635
Anti-LC3B antibody	Abcam	cat# ab192890; RRID:AB_2827794
Goat anti-Rabbit IgG (H + L) Highly Cross-Adsorbed Secondary Antibody, Alexa Fluor^™^ Plus 488	Thermo Fisher	catalog # A32731; RRID:AB_2633280
Donkey anti-Mouse IgG (H + L) Highly Cross-Adsorbed Secondary Antibody, Alexa Fluor^™^ 546	Thermo Fisher	catalog # A10036; RRID:AB_11180613
Donkey anti-Mouse IgG (H + L) Highly Cross-Adsorbed Secondary Antibody, Alexa Fluor^™^ 647	Thermo Fisher	catalog # A-31571; RRID:AB_162542

Biological samples		

Mouse brain tissue	This paper	N/A

Chemicals, peptides, and recombinant proteins		

Ruxolitinib	MedChemExpress	Cat. No.: HY-50856
GSK-7975A	MilliporeSigma	Cat. No.: 5343510001
TRI Reagent	MilliporeSigma	Cat. No.: T9424
iScript^™^ cDNA Synthesis Kit	Bio-Rad	Cat. No.: 1708890
iTaq Universal SYBR Green Supermix	Bio-Rad	Cat. No.: 1725120

Deposited data		

RNA-seq dataset	This paper	GSE300185

Experimental models: Organisms/strains		

*Sting1* N153S Lox-STOP-Lox	Jonathan J Miner (University of Pennsylvania)^18^	Strain #:039266 RRID:IMSR_JAX:039266
B6.129-Tg(Pcp2-cre)2Mpin/J	The Jackson Laboratory	Strain #:004146 RRID:IMSR_JAX:004146
B6(Cg)-*Ifnar1^tm1-2Ees^*/J	The Jackson Laboratory	Strain #:028288 RRID:IMSR_JAX:028288
C57BL/6J mice	The Jackson Laboratory	Strain #:000664 RRID:IMSR_JAX:000664
*Sting1* −/−	Glen N Barber (University of Miami)^37^	N/A

Oligonucleotides		

*m*-Ifnb1 Fwd: 5' -GTCCTCAACTGCTCTCCACT-3'	Sigma-Aldrich	Customized
*m*-Ifnb1 Rev: 5' -CCTGCAACCACCACTCATTC-3'	Sigma-Aldrich	Customized
*m*-Ifit1 Fwd: 5' -TTCACATGGA AGCTGCTATT TGAAA-3'	Sigma-Aldrich	Customized
*m*-Ifit1 Rev: 5' -TGCTC AGCTGCTCGC TCTGGATCAA-3'	Sigma-Aldrich	Customized
*m*-Cxcl10 Fwd: 5' -GGGATCCCTCTCGCAAGGACGGTCC-3'	Sigma-Aldrich	Customized
*m*-Cxcl10 Rev: 5' -ACGCTTTCATTAAATTCTTGATGGT-3 '	Sigma-Aldrich	Customized
*m*-Oas1a Fwd: 5' -GGATGGCATAGATTCTGGGA-3'	Sigma-Aldrich	Customized
*m*-Oas1a Rev: 5' -CTGCATCAGGAGGTGGAGTT-3'	Sigma-Aldrich	Customized
*m*-Bst2 Fwd: 5' -CAAACTCCTGCAACCTGACCGT-3 '	Sigma-Aldrich	Customized
*m*-Bst2 Rev: 5' -CTCCTGGTTCAGCTTCGTGACT-3 '	Sigma-Aldrich	Customized
*m*-Usp18 Fwd: 5' -GTGTCCGTGATCTGGTCCTT-3'	Sigma-Aldrich	Customized
*m*-Usp18 Rev: 5' -CTGCAGAAATACAACGTGCC-3 '	Sigma-Aldrich	Customized
*m*-If¡27 Fwd: 5' -ACATCATTGGATTCGGTTCCTG-3'	Sigma-Aldrich	Customized
*m*-If¡27 Rev: 5' -TGGAGACAATGGAAGCCACC-3 '	Sigma-Aldrich	Customized
*m*-If¡30 Fwd: 5' -GGATAAGCTGGAAAAGGAGGCAG-3 '	Sigma-Aldrich	Customized
*m*-If¡30 Rev: 5' -TCTGGTGACACCTCAGGAGCAT-3 '	Sigma-Aldrich	Customized
*m*-If¡44 Fwd: 5' -AACTGACTGCTCGCAATAATGT-3'	Sigma-Aldrich	Customized
*m*-If¡44 Rev: 5' -GTAACACAGCAATGCCTCTTGT-3 '	Sigma-Aldrich	Customized
*m*-Rsad2 Fwd: 5' -GGACGCTTCATGGTGTTATTTG-3'	Sigma-Aldrich	Customized
*m*-Rsad2 Rev: 5' -TGATTGGTCGCCTGTTTATCT-3 '	Sigma-Aldrich	Customized
m-Il6 Fwd: 5' -CACAAGTCCGGAGAGGAGAC-3'	Sigma-Aldrich	Customized
m-Il6 Rev: 5' -CAGAATTGCCATTGCACAAC-3'	Sigma-Aldrich	Customized
*m*-Tnf Fwd: 5' -CTACCTTGTTGCCTCCTCTTT-3 '	Sigma-Aldrich	Customized
*m*-Tnf Rev: 5' -GAGCAGAGGTTCAGTGATGTAG-3 '	Sigma-Aldrich	Customized
m-Il1a Fwd: 5' -CGAAGACTACAGTTCTGCCATT-3 '	Sigma-Aldrich	Customized
m-Il1a Rev: 5' -GACGTTTCAGAGGTTCTCAGAG-3'	Sigma-Aldrich	Customized
m-Il1b Fwd: 5' -GCTTCCTTGTGCAAGTGTCT-3'	Sigma-Aldrich	Customized
m-Il1b Rev: 5' -GGTGGCATTTCACAGTTGAG-3'	Sigma-Aldrich	Customized
m-Il18 Fwd: 5' -GACTCTTGCGTCAACTTCAAGG-3'	Sigma-Aldrich	Customized
m-Il18 Rev: 5' -CAGGCTGTCTTTTGTCAACGA-3'	Sigma-Aldrich	Customized
*m*-Cxcl1 Fwd: 5' -GCTTGAAGGTGTTGCCCTCAG-3 '	Sigma-Aldrich	Customized
*m*-Cxcl1 Rev: 5' -AAGCCTCGCGACCATTCTTG-3 '	Sigma-Aldrich	Customized
*m*-Cxcl2 Fwd: 5' -GCGCTGTCAATGCCTGAAGA-3 '	Sigma-Aldrich	Customized
*m*-Cxcl2 Rev: 5' -TTTGACCGCCCTTGAGAGTG-3 '	Sigma-Aldrich	Customized
*m*-Ccl4 Fwd: 5' -AAACCTAACCCCGAGCAACA-3 '	Sigma-Aldrich	Customized
*m*-Ccl4 Rev: 5' -CCATTGGTGCTGAGAACCCT-3 '	Sigma-Aldrich	Customized
*m*-Ccl5 Fwd: 5' -ACTCCCTGCTGCTTTGCCTAC-3 '	Sigma-Aldrich	Customized
*m*-Ccl5 Rev: 5' -ACTTGCTGGTGTAGAAATACT-3 '	Sigma-Aldrich	Customized
*m*-Ccl7 Fwd: 5' -CAGAAGGATCACCAGTAGTCGG-3 '	Sigma-Aldrich	Customized
*m*-Ccl7 Rev: 5' -ATAGCCTCCTCGACCCACTTCT-3'	Sigma-Aldrich	Customized
*m*-Ccl8 Fwd: 5' -AAGCTGACTGGGCCAGATAAGGCTC-3'	Sigma-Aldrich	Customized
*m*-Ccl8 Rev: 5' -CAAGGATCTCCATGTACTCACTGAC-3 '	Sigma-Aldrich	Customized
*m*-Ccl11 Fwd: 5' -GATCTTCTTACTGGTCATGATAAAGCA-3'	Sigma-Aldrich	Customized
*m*-Ccl11 Rev: 5' -TGTCTCCCTCCACCATGCA-3 '	Sigma-Aldrich	Customized
*m*-Gapdh Fwd: 5' -TTCACCACCATGGAGAAGGC-3'	Sigma-Aldrich	Customized
*m*-Gapdh Rev: 5' -GGCATCGACTGTGGTCATGA-3'	Sigma-Aldrich	Customized
*m*-Hprt Fwd: 5' -ATGTCATGAAGGAGATGGGAGGCCA-3'	Sigma-Aldrich	Customized
*m*-Hprt Rev: 5' -TCTCCACCAATAACTTTTATGTCCC-3 '	Sigma-Aldrich	Customized
*m*-Actb Fwd: 5' -TTCTTTGCAGCTCCTTCGTT-3'	Sigma-Aldrich	Customized
*m*-Actb Rev: 5' -ATGGAGGGGAATACAGCCC-3'	Sigma-Aldrich	Customized

Software and algorithms		

GraphPad Prism	GraphPad Software	RRID:SCR_002798
ZEISS ZEN Microscopy Software	ZEISS	RRID:SCR_013672
Adobe Photoshop	Adobe	RRID:SCR_014199
QuPath	QuPath	RRID:SCR_018257
Fiji	NIH	RRID:SCR_002285
Axopatch 200B Patch Clamp Amplifier	AutoMate Scientific, Inc.	RRID:SCR_018866
pClamp-10 software	Molecular Devices	RRID:SCR_011323

Other		

Allen Brain Map Transcriptomics Explorer	Allen Institute	N/A
Mouse Brain Atlas	http://mousebrain.org/	N/A

## Highlights

- STING is expressed in Purkinje cells in the cerebellum
- STING activation in Purkinje cells leads to neurodegeneration and cerebellar ataxia
- STING signaling in Purkinje cells reduces autonomous firing frequency
- STING-mediated neurodegeneration in Purkinje cells is IFN-independent
