# Supplementary material for: Autonomous STING signaling in Purkinje cells drives neurodegeneration independent of type I interferon
Source: Cell Rep. Author manuscript; Available in PMC 2025 Dec 29. (PMC12746686; doi:10.1016/j.celrep.2025.116480)
Supplement: 1 [file NIHMS2125810-supplement-1.pdf]

Cell Reports, Volume *44*

Supplemental information

Autonomous STING signaling

in Purkinje cells drives neurodegeneration

independent of type I interferon

Kun Yang, Miranda Dunn, Gustavo Torres-Ramirez, Nicole Dobbs, Vikram G. Shakkottai, and Nan Yan

Supplementary figures

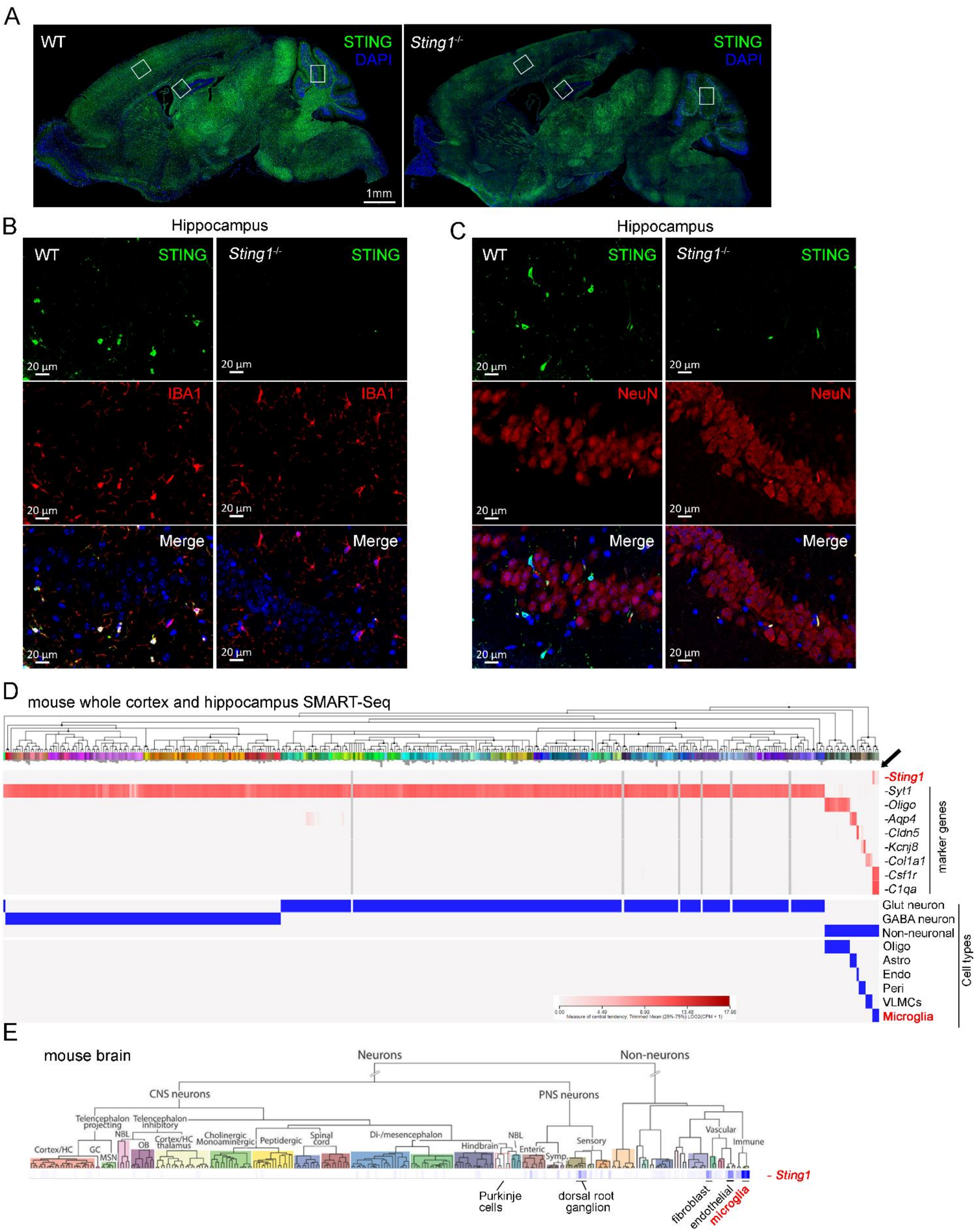

Figure S1. ***Sting1*** **expression in brain cells.**

(**A**) Representative immunofluorescence images of STING protein (green) in WT and *Sting1*$^{-/-}$ mouse brains. Note: opposite hemispheres were sectioned in two genotypes and *Sting1*$^{-/-}$ image was mirrored horizontally for visualization. (**B**, **C**) Representative immunofluorescence images of STING protein (green) in microglia (Iba1), neurons (NeuN) in hippocampi of WT and *Sting1*$^{-/-}$ mice. (**D**) Heat map of *Sting1* and cell type maker genes mRNA expression in different brain cell types. Data are from the whole mouse cortex and hippocampus SMART-seq available at Allen Brain Map Transcriptomics Explorer. (**E**) Heat map of *Sting1* mRNA expression in different brain cell types. Data are from scRNA-seq of the adolescent mouse brain available at http://mousebrain.org/.

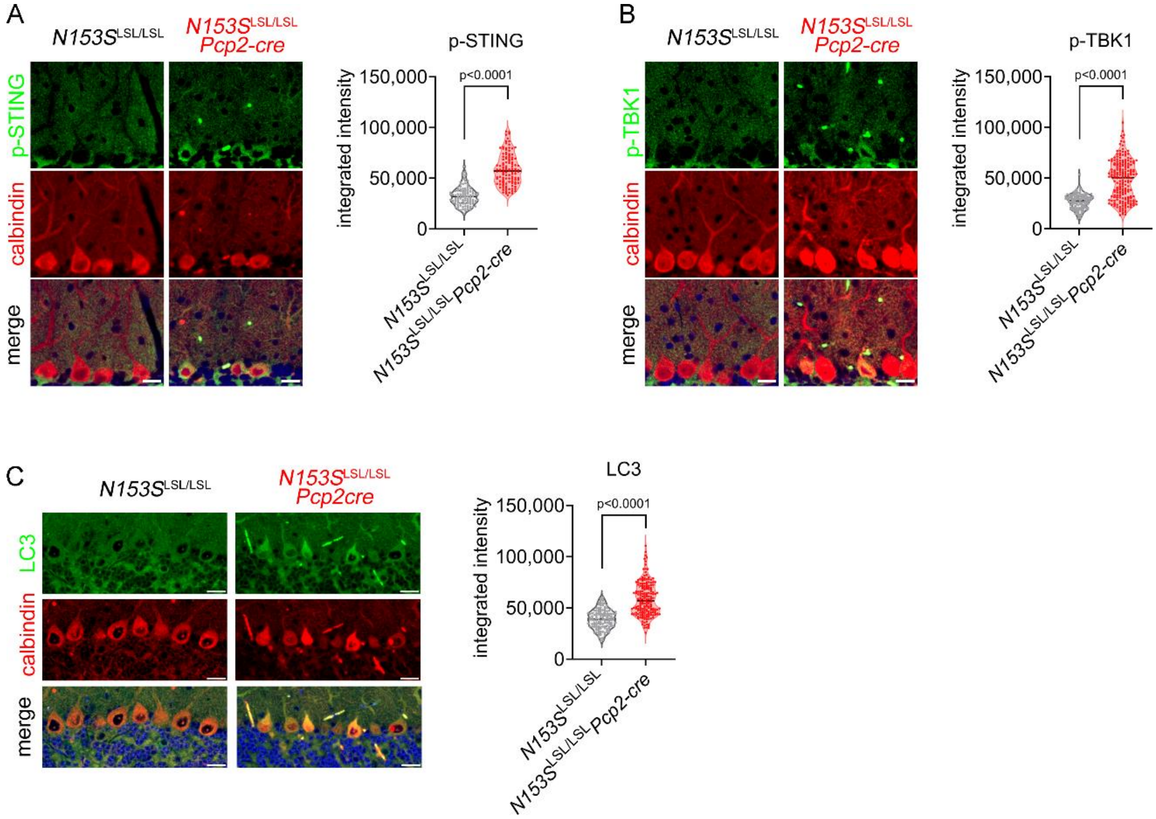

Figure S2. Activation of STING signaling in $\boldsymbol{N153S^{\mathrm{LSL/LSL}}}$***Pcp2-cre*** **Purkinje cells.**

(**A**) Representative immunofluorescence images of p-STING (green) in Purkinje cells (calbindin in red) of 1-month-old $N153S^{\mathrm{LSL/LSL}}$ and $N153S^{\mathrm{LSL/LSL}}$*Pcp2-cre* mice. Quantification of p-STING intensity in Purkinje cell soma is shown on the right (more than 110 randomly selected Purkinje cells from 4 mice per genotype). Data are shown as mean ± SEM; two-tailed unpaired t-test. (**B**) Representative immunofluorescence images of p-TBK1 (green) in Purkinje cells (calbindin in red) of 1-month-old $N153S^{\mathrm{LSL/LSL}}$ and $N153S^{\mathrm{LSL/LSL}}$*Pcp2-cre* mice. Quantification of p-STING intensity in Purkinje cell soma is shown on the right (more than 140 randomly selected Purkinje cells from 4 mice per genotype). Data are shown as mean ± SEM; two-tailed unpaired t-test. (**C**) Representative immunofluorescence images of LC3 (green) in Purkinje cells (calbindin in red) of 1-month-old $N153S^{\mathrm{LSL/LSL}}$ and $N153S^{\mathrm{LSL/LSL}}$*Pcp2-cre* mice. Quantification of p-STING intensity in Purkinje cell soma is shown on the right (more than 130 randomly selected Purkinje cells from 4 mice per genotype). Data are shown as mean ± SEM; two-tailed unpaired t-test.

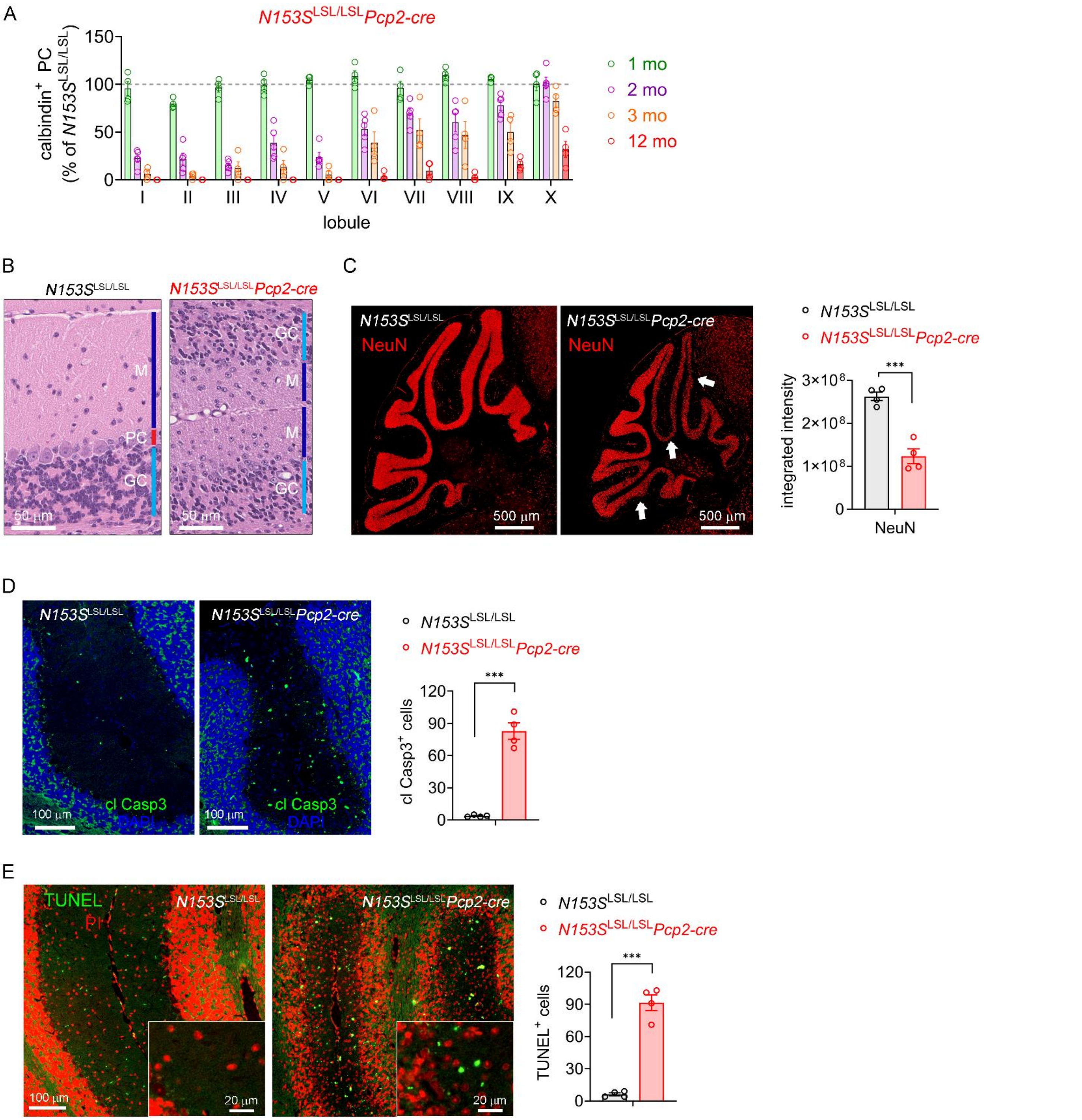

Figure S3. Cerebellar degeneration of *N153S*$^{\text{LSL/LSL}}$*Pcp2-cre* mice.

(**A**) Quantification of calbindin+ Purkinje cells by lobules. The percentage of calbindin+ Purkinje cells in *N153S*$^{\text{LSL/LSL}}$*Pcp2-cre* mice relative to *N153S*$^{\text{LSL/LSL}}$ controls in the corresponding lobules is shown. (**B**) Representative cerebellar H&E staining of 12-month-old *N153S*$^{\text{LSL/LSL}}$*Pcp2-cre* and *N153S*$^{\text{LSL/LSL}}$ littermate mice. M, molecular layer, PC, Purkinje cell layer, GC, granule cell layer. (**C**) Representative

immunofluorescence images of granule cells (NeuN in red) in 12-month-old *N153S*$^{LSL/LSL}$*Pcp2-cre* and *N153S*$^{LSL/LSL}$ mouse brain. Nuclei were stained with DAPI in blue. Quantification of NeuN integrated intensity in granule layer is shown on the right. Data are shown as mean ± SEM (n=4 mice per genotype). Two-tailed unpaired t-test, *** $p < 0.001$. (**D**) Representative cleaved caspase 3 (cl Casp3, in green) immunostaining of 12-month-old *N153S*$^{LSL/LSL}$*Pcp2-cre* and *N153S*$^{LSL/LSL}$ mouse brains. Nuclei were stained with DAPI in blue. Quantification of cl Casp3+ cells in cerebellar cortex per mid-sagittal section is shown on the right. Data are shown as mean ± SEM (n=4 mice per genotype). Two-tailed unpaired t-test, *** $p < 0.001$. (**E**) Representative TUNEL (in green) staining of 12-month-old *N153S*$^{LSL/LSL}$*Pcp2-cre* and *N153S*$^{LSL/LSL}$ mouse brain. Nuclei were stained with propidium iodide in red. Quantification of TUNEL+ cells in cerebellar cortex per mid-sagittal section is shown on the right. Data are shown as mean ± SEM (n=4 mice per genotype). Two-tailed unpaired t-test, *** $p < 0.001$.

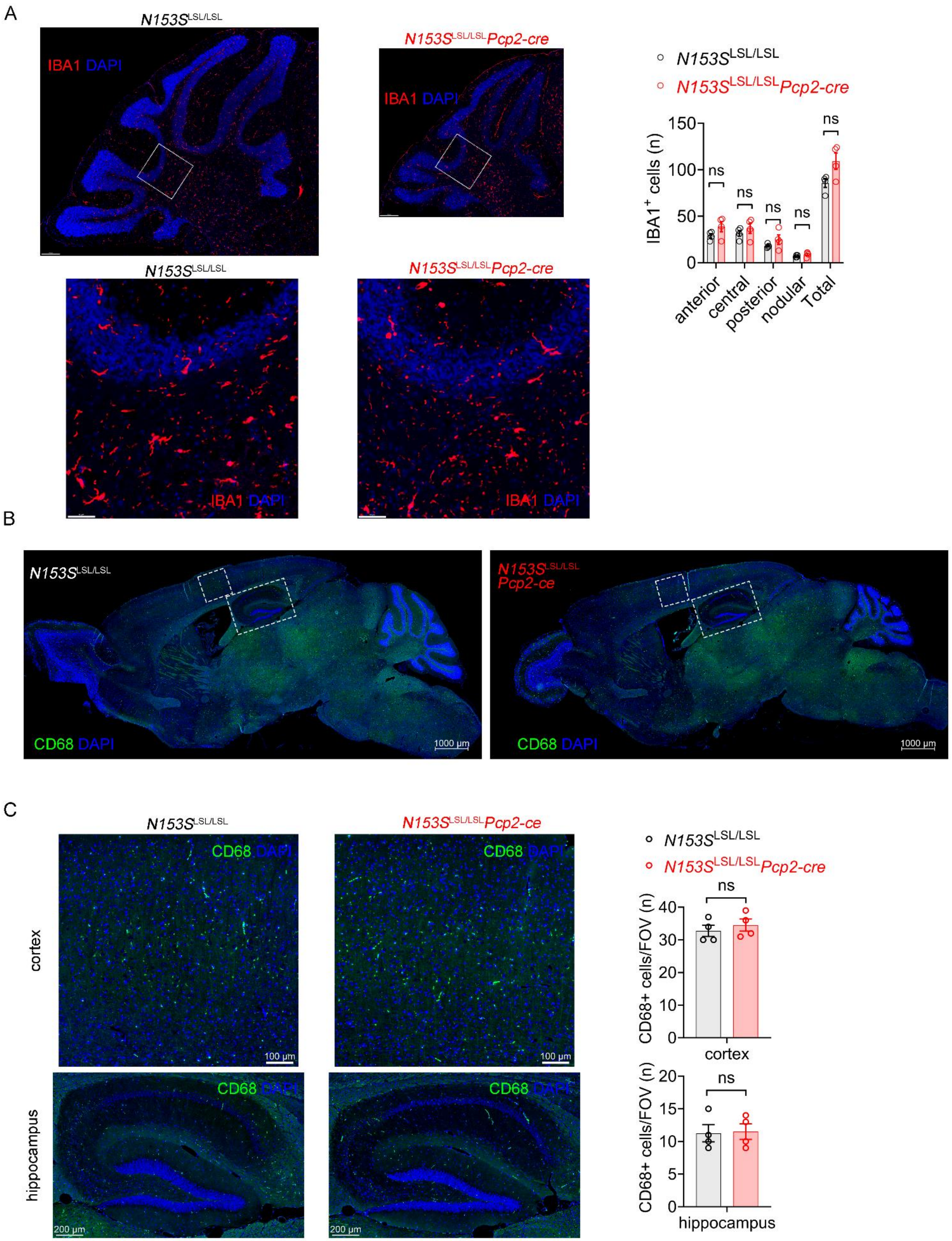

Figure S4. IBA1 and CD68 staining of *N153S*$^{LSL/LSL}$*Pcp2-cre* cerebellum.

(**A**) Representative immunofluorescence images of microglia marker IBA1 (red) in 12-month-old *N153S*$^{LSL/LSL}$*Pcp2-cre* and *N153S*$^{LSL/LSL}$ mouse brains. Nuclei were stained with DAPI in blue. Note: *N153S*$^{LSL/LSL}$*Pcp2-cre* image is smaller because of cerebellar atrophy in brains of these mice. Quantification of IBA1 positive microglia in anterior, central, posterior and nodular lobes. Data are shown as mean ± SEM (n=4 mice per genotype). ns, not significant by two-tailed unpaired *t*-test. (**B, C**) Representative immunofluorescence images of microglia activation marker CD68 (green) in 12-month-old *N153S*$^{LSL/LSL}$*Pcp2-cre* and *N153S*$^{LSL/LSL}$ mouse brains. Nuclei were stained with DAPI in blue. Quantification of CD68 positive cells in cerebral cortex and hippocampus is shown in bar graphs. Data are shown as mean ± SEM (n=4 mice per genotype). ns, not significant by two-tailed unpaired *t*-test.

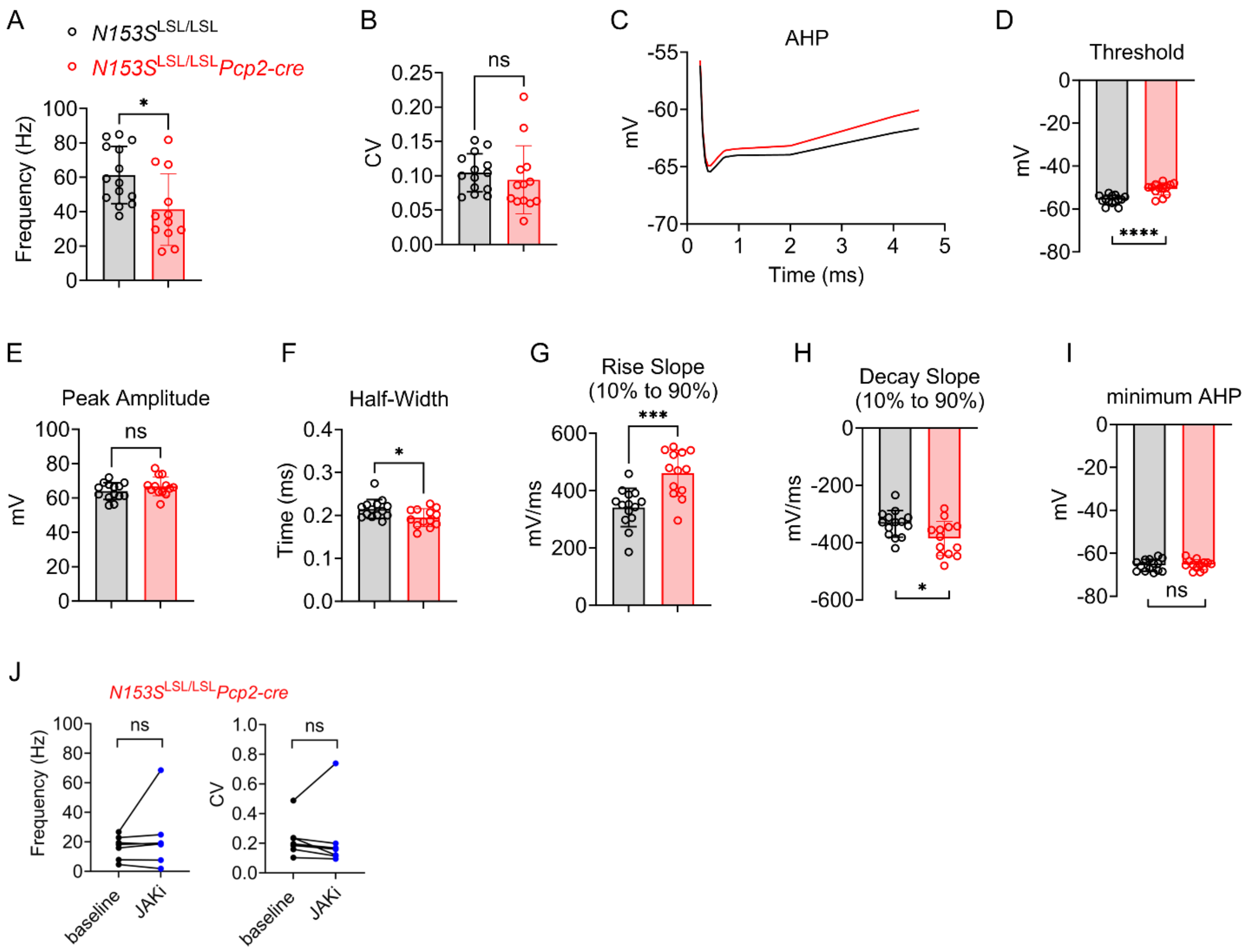

Figure S5. Whole-cell electrophysiology of *N153S*$^{LSL/LSL}$*Pcp2-cre* Purkinje cells

(**A**) Analysis of firing frequency of Purkinje cells in *N153S*$^{LSL/LSL}$ (n=14 cells) and *N153S*$^{LSL/LSL}$*Pcp2-cre* (n=12 cells) mice recorded in patch-clamp whole-cell configuration using an unpaired t-test. (**B**) Analysis of the coefficient of variation of Purkinje cells in *N153S*$^{LSL/LSL}$ (n=14 cells) and *N153S*$^{LSL/LSL}$*Pcp2-cre* (n=13 cells) mice recorded in patch-clamp whole-cell configuration using an unpaired t-test. (**C**) Average AHP trace starting from the peak of the action potential. (**D**) Threshold of action potentials in Purkinje cells in *N153S*$^{LSL/LSL}$ (n=14 cells) and *N153S*$^{LSL/LSL}$*Pcp2-cre* (n=13 cells) mice recorded in patch-clamp whole-cell configuration using an unpaired t-test. (**E**) Analysis of the peak amplitude of action potentials in Purkinje cells in *N153S*$^{LSL/LSL}$ (n=14 cells) and *N153S*$^{LSL/LSL}$*Pcp2-cre* (n=13 cells) mice using an unpaired t-test. (**F**) Analysis of the half-width of action potentials in Purkinje cells in *N153S*$^{LSL/LSL}$ (n=14 cells) and *N153S*$^{LSL/LSL}$*Pcp2-cre* (n=13 cells) mice using an unpaired t-test. (**G, H**) Analysis of the rise and decay slopes of action potentials in Purkinje cells in *N153S*$^{LSL/LSL}$ (n=14 cells) and *N153S*$^{LSL/LSL}$*Pcp2-cre* (n=13 cells) mice using an unpaired t-test. (**I**) Analysis of minimum AHP in Purkinje cells in *N153S*$^{LSL/LSL}$ (n=14 cells) and *N153S*$^{LSL/LSL}$*Pcp2-cre* (n=13 cells) mice using an

unpaired t-test. *p<0.05, ***p<0.001, ****p<0.0001, ns, not significant. (**J**) Firing frequency and coefficients of variation of *N153S*$^{LSL/LSL}$*Pcp2-cre* Purkinje cells (n=6) treated with JAK inhibitor Ruxolitinib (JAKi, 20 μM) analyzed with a paired t-test.
